# MicroRNA Transcriptome in Swine Small Intestine during Weaning Stress

**DOI:** 10.1371/journal.pone.0079343

**Published:** 2013-11-18

**Authors:** Xin Tao, Ziwei Xu

**Affiliations:** Institute of Animal Husbandry and Veterinary Science, Zhejiang Academy of Agricultural Sciences, Hangzhou, Zhejiang Province, China; Harbin Institute of Technology, China

## Abstract

MicroRNAs (miRNAs) play important roles in intestinal diseases; however, the role of miRNAs during weaning stress is unknown. In our study, six jejunal small RNA libraries constructed from weaning piglets at 1, 4 and 7 d after weaning (libraries W1, W4 and W7, respectively) and from suckling piglets on the same days as the weaning piglets (libraries S1, S4 and S7, respectively) were sequenced using Solexa high-throughput sequencing technology. Overall, 260 known swine miRNAs and 317 novel candidate miRNA precursors were detected in the six libraries. The results revealed that 16 differentially expressed miRNAs were found between W1 and S1; 98 differentially expressed miRNAs were found between W4 and S4 (ssc-mir-146b had the largest difference); and 22 differentially expressed miRNAs were found between W7 and S7. Sequencing miRNA results were validated using RT-qPCR. Approximately 11,572 miRNA-mRNA interactions corresponding to 3,979 target genes were predicted. The biological analysis further describe that the differentially expressed miRNAs regulated small intestinal metabolisms, stressful responses and immune functions in piglets. Therefore, the small intestine miRNA transcriptome was significantly different between weaning and suckling piglets; the difference varied with the number of days after weaning.

## Introduction

MicroRNAs (miRNAs) belong to a group of endogenous small non-coding RNAs that are 18–26 nucleotides (nt) in length. In animals, almost all miRNAs regulate gene expression by binding complementary target sites in the 3′untranslated region (3′ UTR) of mRNA [1]. More than one-third of all protein-coding genes in humans are regulated by miRNAs [2]. Studies have shown that miRNAs play major roles in cellular growth, differentiation, proliferation, apoptosis and immune response [3]–[6]. Even though miRNAs are involved in the ‘fine tuning’ of gene expression, the functions of miRNAs are more evident during disease [7].

Weaning stress affects intestinal morphology, digestive and metabolic enzymes and the intestinal mucosal barrier. In the past years, numerous studies reported small intestinal villus atrophy and crypt hyperplasia in piglets [8]–[10]. Weaning disturbed digestive enzyme activities and inflammatory cytokine gene expression patterns of the intestine, impaired intestinal barrier function, increased intestinal permeability, and promoted the bacteria, toxins and antigens to pass through the intestinal epithelium [11]–[13]. Furthermore, weaning also activated stress signaling pathways and resulted in an abnormal expression of intestinal genes and proteins [14]–[18]. The most serious effects of weaning on intestinal tissue took place at the first 1 week after weaning [19], [20]. However, the cellular and molecular mechanisms associated with weaning stress on small intestine have not been elucidated. In recent years, more and more miRNAs have been discovered in the small intestinal tissue and cells of mammals [21]–[24]. Certain intestinal miRNAs have been identified in a tissue-specific manner [25], [26].

According to reports, miRNAs control intestinal cell differentiation, physiology, barrier function and cellular apoptosis [27], [28]. Furthermore, miRNAs are involved in the pathogenesis of intestinal cancer, inflammatory bowel disease, irritable bowel syndrome and cystic fibrosis, among others [29]–[33]. Therefore, the miRNA transcriptome of small intestine is likely to be affected by weaning. However, no study has focused on the miRNA expression levels in the small intestine during weaning stress. Swine have been used as a mammalian model of humans due to similarities in size, physiology, organ development and disease progression [34]. Similar to weaning in piglets, weaning in infants is a critical point due to changes in intestinal homeostasis [35].

To assess the effects and mechanism of differentially expressed miRNAs on intestinal damage during weaning stress, we compared the intestinal miRNA transcriptome of weaning and suckling piglets using Solexa high-throughput sequencing technologies. Six small RNA libraries were prepared from jejunal samples: W1, W4 and W7 prepared from samples obtained on days 1, 4 and 7 after weaning, respectively, and S1, S4 and S7 (controls) prepared from samples obtained from suckling piglets on the same days on which the weaning samples were collected. Each library was sequenced individually and generated several million sequence reads, resulting in a total of more than 34 million reads. Statistical analyses were performed using Rfam10.1 and miRBase19.0 databases. BLAST and miRDeep methods were used for aligning known miRNAs and discovering novel miRNA candidate precursors from the high-throughput sequencing results.

## Results

### Identification and Mapping of miRNAs

The reads obtained from Solexa high-throughput sequencing were processed; low-quality data and 3′ and 5′ adapters were removed, and poly-A/T-containing sequences and fragments <15 nt and >32 nt were discarded (Table S1). The remaining clean reads were 84.32% and 83.65% in W1 and S1, 74.34% and 82.76% in W4 and S4, and 81.28% and 81.93% in W7 and S7, respectively. The results revealed that there were no dramatic differences in the clean reads among the libraries.

The small RNA length distribution in the libraries is shown in Figure S1. The majority of the sequences (82.57–88.29%) were 20–23 nt in size. The most abundant read was 21 nt, followed by 22 nt and 20 nt. Of the total reads, the 21 nt read was 36.98% and 40.24% in W1 and S1, 29.45% and 40.55% in W4 and S4, and 33.29% and 39.79% in W7 and S7, respectively. The miRNA length distribution was typical of animal miRNAs. The results suggested that miRNA sequences were highly enriched in the libraries. Interestingly, in the weaning groups, the percentage of the 21 nt reads was lower and the percentage of the 22 nt reads was higher than in the control (suckling) groups; the difference was more significant between W4 and S4 than between the other two pairs.

Clean data were mapped to the *Sus scrofa* draft genome assembly (SGSC Sscrofa 9.2, NCBI project 10718, GCA_000003025.2) with no mismatches, using the Bowtie program (Table S2 and Table S3). The mapped reads were 3,583,379 and 3,365,491 in W1 and S1, 3,852,427 and 3,970,888 in W4 and S4, and 3,688,395 and 3,967,028 in W7 and S7, respectively. Of the total reads, the mapped reads accounted for 80.57% and 80.12% in W1 and S1, 80.85% and 81.10% in W4 and S4, and 82.04% and 80.28% in W7 and S7, respectively. The results showed the distribution of reads was not equal on the swine chromosomes. The most reads were mapped to SSC2, and the values were between 1,782,073 and 2,229,151. In the next place, the reads mapped to SSC10 were 230,821∼695,256 in the form of antisense or sense reads, respectively. However, subsequent miRNAs identified in the libraries showed that the maximum numbers of miRNAs were not located on SSC2 or SSC10, suggesting that most of sequencing data acquired from these chromosomes was not significant for detecting miRNAs. To confirm the counts and proportions of miRNAs, the clean reads were aligned to the miRNA precursors and mature miRNAs in miRbase19.0 (Table S4 and Table S5). The results showed that approximately 74.07–77.95% of clean reads in six libraries were matched to the miRNA precursors; however, unique reads were 5.71–8.30%. Similarly, 69.75–73.68% of clean reads in six libraries were matched to the mature miRNAs; however, unique reads were 3.22–4.93% (Table S6). In addition, the miRNA precursors and mature miRNAs in W4 had more abundant species than the precursors and mature miRNAs in the other libraries.

We chose the Rfam10.1 database for annotating small RNA reads and unique reads (Figure S2 and Figure S3). The results revealed that there were no significant differences in the distribution of reads among the six libraries. The most abundant class of small non-coding RNAs (ncRNAs) was rRNA (small nuclear RNA (snRNA)/small nucleolar (snoRNA)/CD-box and snRNA/splicing).

### Identification of Known and Novel miRNA Candidate Precursors

To identify known swine miRNAs, we compared the clean reads obtained in this study with 271 known swine miRNA precursors in miRBase19.0 (Table S7). The statistical results showed that 260 known unique miRNA precursors were detected from the read counts in our libraries. Only 11 miRNAs (ssc-miR-1224, ssc-miR-202, ssc-miR-4333, ssc-miR-4335, ssc-miR-4336, ssc-miR-4338, ssc-miR-4339, ssc-miR-484, ssc-miR-487b, ssc-miR-489 and ssc-miR-494) were not detected, which may be due to low or absent expression in swine intestine. The species accounted for 95.94% of all swine miRNA precursors. In addition, there were 126 mature miRNAs distinctively derived from the 5 p arm and 3 p arm of the 63 precursors (Table S8). The known and novel miRNA candidate precursors had a very wide span of expression, so that the read counts ranged from one to millions.

**Figure 1:**
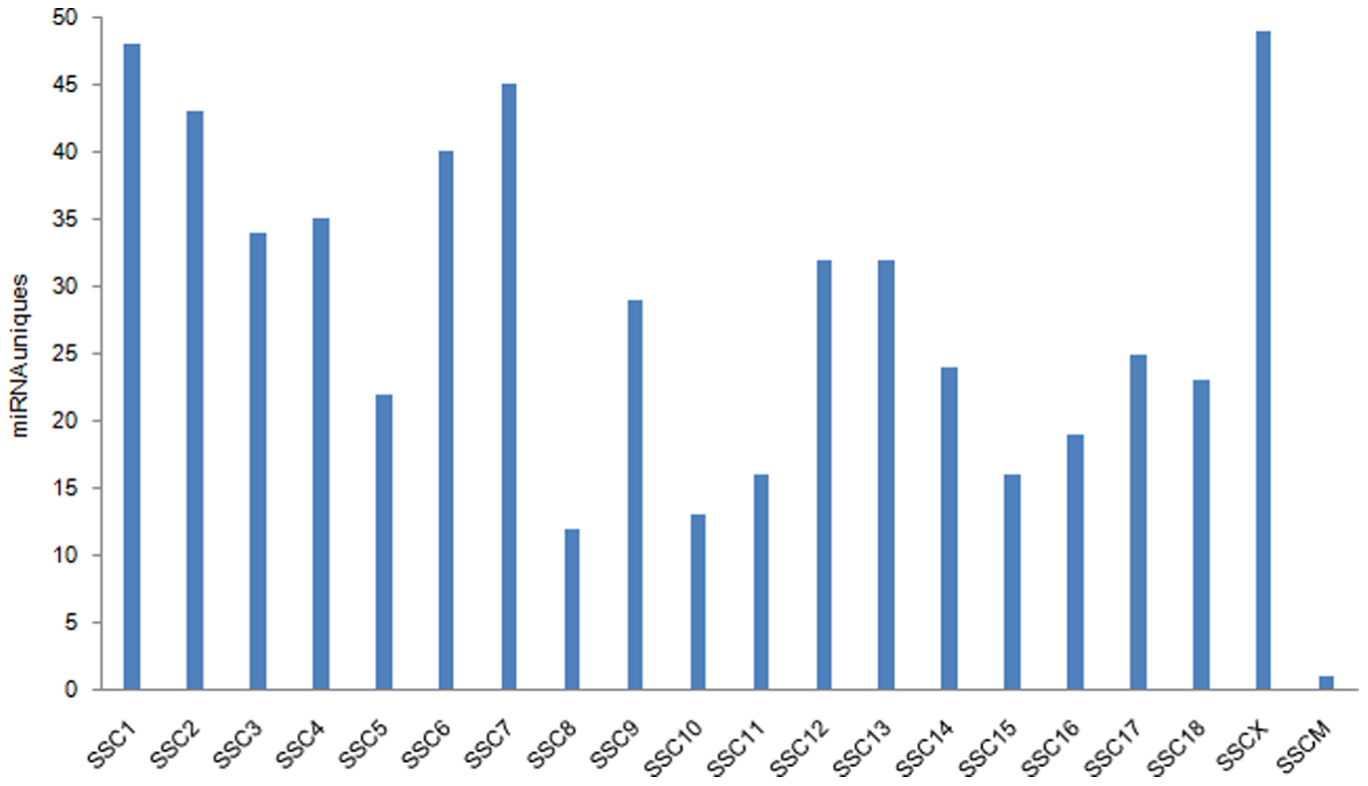
Distribution of novel candicate miRNAs in Chromosomes. The y-axis is the number of novel candidate miRNA uniques predictecd in six libraries (W1, W4, W7, S1, S4, S7). W1, W4 and W7, the samples from piglets on days1, 4 and 7 after weaning, respectively. S1, S4 and S7, the samples from suckling piglets on the same days on which the weaning samples were collected.

We identified 317 novel miRNA candidate precursors in the libraries (Table S9), in which 260 candidates shared the same seed sequences with known miRNA names across species, such as 77 candidates in mouse and 53 candidates in human, etc. Other 57 novel miRNA candidate precursors have no same seeds with other species, which are likely pig-specific miRNAs, or not bona fide miRNAs. The distributions of novel miRNAs on different chromosomes are shown in Figure 1. Novel miRNA candidate precursors were detected on each chromosome. The maximum number of miRNAs was located on SSC1, followed by SSC7 and SSC2. The minimum number of miRNAs was located on SSC8. Additionally, we found one miRNA candidate precursor on SSCM (mitochondrion). The results revealed that an unequal distribution of miRNAs on the chromosomes would probably explain miRNA functions in the small intestine.

### Analysis of Differentially Expressed Swine miRNAs

**Figure 2:**
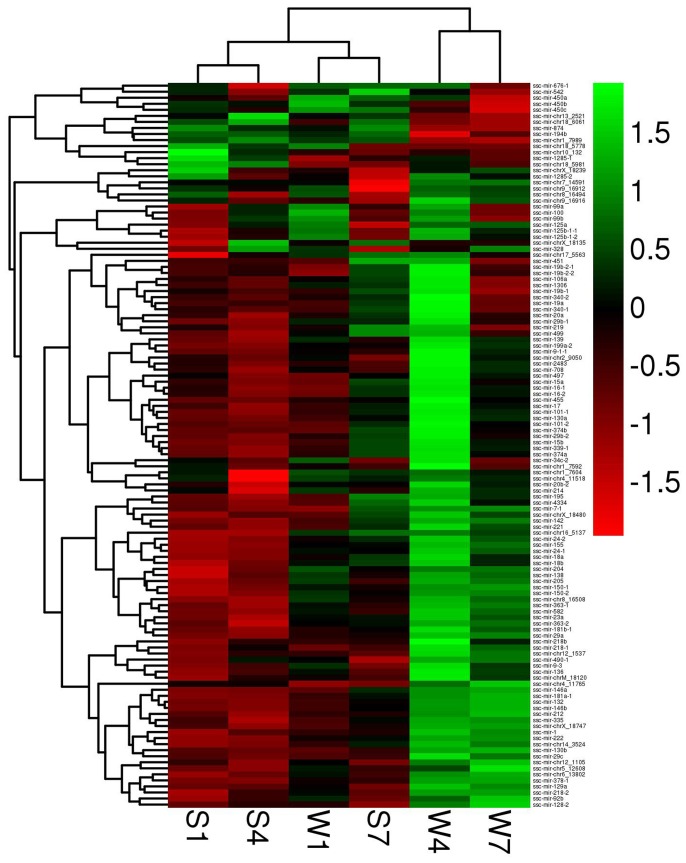
Heatmap of differentially expressed miRNAs in weaning and control groups. W1, W4 and W7, the samples from piglets on days1, 4 and 7 after weaning, respectively. S1, S4 and S7, the samples from suckling piglets on the same days on which the weaning samples were collected. Color levels were normalized row scales and the values of scale were the log10(FPKM+1) values transformed by the FPKMs of miRNAs. Green indicates upregulated expression, and red indicates downregulated expression compared to a reference expression level.

We compared differentially expressed miRNAs in W1 and S1, W4 and S4, and W7 and S7 (Table S10 and Figure 2). We found that 16 miRNAs had different expression levels between W1 and S1; of these 16 miRNAs, 11 were upregulated and 5 were downregulated in the weaning group. Likewise, 98 differentially expressed miRNAs were found between W4 and S4; 92 upregulated miRNAs and 6 downregulated miRNAs were detected in the weaning group. Finally, there were 22 differentially expressed miRNAs between W7 and S7; 15 upregulated miRNAs and 7 downregulated miRNAs in the weaning group. However, no differentially expressed miRNAs were found to overlap across the three time points. Only 3 miRNAs (miR-155, miR-150-1 and miR-204) at W1 and W4, and 4 miRNAs (miR-132, miR-146b, miR-212 and miR-218-2) at W4 and W7 were consistently upregulated across two of three time points. This may be concerned with miRNA sequential-specific expression and/or be affected by different physiological response. Anyway, our results revealed that weaning significantly affected miRNAs expression levels in the small intestine. Furthermore, the effects varied with the number of days after weaning. The largest difference in miRNA expression levels were obtained between W4 and S4. Therefore, subsequent analyses focused on W4 and S4. In addition, miR-215 and miR-146b out of all differentially expressed miRNAs were particularly interested, because the former had the abundant expression levels in all libraries and was one of several miRNAs that had a downregulated trend in the weaning groups, and the latter had the largest differences in expression levels between two pairs of W4 and S4, W7 and S7.

### Validation of High-throughput Sequencing Results by Real-time Quantitative PCR

**Figure 3:**
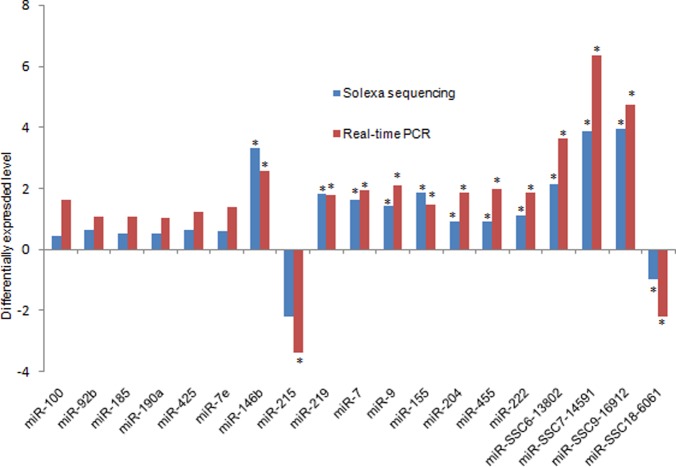
Validation of sequencing results by real-time quantitative PCR. Four biological replicates were used, and U6 snRNA was used as an internal control. The y-axis is the fold-change between W4 and S4 samples (fold quantity values for real-time PCR; Log (W4/S4, 2) for Solexa sequencing). Means with the star on the column differ significantly (P<0.05). W4, the samples from piglets on days 4 after weaning. S4, the samples from suckling piglets on the same days on which W4 samples were collected.

To validate the Solexa high-throughput sequencing results, 15 known miRNAs and 4 novel miRNA candidate precursors were randomly selected from W4 and S4 to quantify their relative expression levels. The miRNA primers are shown in Table S11. The expression levels of miRNAs obtained from sequencing and from real-time quantitative polymerase chain reaction (RT-qPCR) were compared (Figure 3). Almost all miRNA expression levels were consistent between the two methods. The expression levels of ssc-miR-215 were significantly different between W4 and S4 (p<0.05) based on the RT-qPCR results. On the other hand, the sequencing results revealed no significant differences (q >0.05) in the expression levels of ss-miR-215 between the two groups. However, the corrected q-value obtained by the sequencing results was 0.055, which was close to q <0.05. Differences in the results were probably attributable to biological differences in the samples. We validated the different expression levels of four novel miRNA candidate precursors by RT-qPCR.

### Gene Target Prediction and Functional Annotation

The potential target genes of differentially expressed miRNAs in W4 and S4 were predicted using the Targetscans software [36]. In total, 11,572 miRNA-mRNA interaction sites corresponding to 3,979 target genes were predicted for the 98 differentially expressed miRNAs in W4 and S4, in which 3,785 genes were predicted for the 92 upregulated miRNAs and 974 genes for the 6 downregulated miRNAs, only 194 genes were not overlapped for those of downregulated miRNAs (Table S12 and Table S13). Our results shared the same conclusion of Lian et al [37] that showed most of miRNA species regulated scores of target genes; similarly over half of target genes were regulated by more than one miRNA.

We also analyzed the functions of differentially expressed miRNAs by performing Gene Ontology (GO) terms and Kyoto Encyclopedia of Genes and Genomes (KEGG) pathway annotations of the predicted miRNA targets using the GOEAST and DAVID gene annotation tool, respectively. The pathway enrichment lists were shown in Table S14 and Table S15.

## Discussion

In our study, the number of differentially expressional miRNAs in small intestine was very significantly increased at 4 d than at 1 d and 7 d after weaning, suggesting that the stressful injuries to the small intestine took place as soon as weaning, and this disadvantages were deteriorating at 4 d after weaning and gradually alleviating at 7 d after weaning. Our results were consistent with those of previous studies, which reported the most severe damage to porcine intestinal morphology was observed in 2–5 d after weaning [10], [38]. Similar to our results, stress trials have reported intestinal miRNA expression. Infections with *Eimeria papillata* parasites induced miRNA expression in mouse jejunum; four miRNAs (miR-1959, miR-203, miR-21 and miR-M23-1-5p) were upregulated [39]. Researchers, who assessed the effects of garlic on miRNA expression levels in mice, reported that garlic downregulated miR-1959, miR-203 and miR-21 and upregulated 11 miRNAs [40]. In rats suffering from heat stress, 18 miRNAs were upregulated and 11 were downregulated [41]. Microbial colonization affected microRNA expression levels in mouse ileum and colon [42], [43].

In this study, miR-146b out of all miRNAs differentially expressed had the largest range at 4 d after weaning and had a sustaining difference at 7 d after weaning; miR-215 out of all miRNAs detected in all libraries showed the abundant expression level and was one of several downregulated expression miRNAs in the weaning groups. It is suggested that their functions are very important in swine intestine. miR-146b was significantly upregulated during stages I, II and III of papillary thyroid carcinoma [44]. Increasing evidence has revealed that the miR-146 family (miR-146a and miR-146b) mediates IRAK1 (interleukin 1 receptor-associated kinase) and TGF-β (transforming growth factor-β) signaling pathways through negative feedback regulation during intestinal epithelial cell differentiation and in the mucosal immune system [45], [46]. In accordance with our results, miR-215 was highly expressed in the posterior segment of the jejunum of 31-d-old piglets [24]. However, the expression of miR-215 was found to be upregulated in the duodenum of E.coli F18-sensitive pigs [47]. The opposite results were possibly attributed to differences in stress response. Furthermore, miR-215 is thought to be involved in the pathogenesis of colon cancer as a tumor suppressor and/or candidate prognostic gene [48], [49].

In our study, other 6 miRNAs (miR-155, miR-150-1, miR-204, miR-132, miR-212 and miR-218-2) were consistently upregulated across two of three weaning times. However, few reports were available in pigs, more studies focused on these miRNAs in humans. miR-155 was earlier found to play critical roles in various physiological and pathological processes such as immunity, inflammation, cancer, cardiovascular disease and haematopoietic lineage differentiation, and involved in viral infections [50]. The mechanisms of miR-155 on host inflammation and immunity were demonstrated to be associated with regulating the TLR3/TLR4 (toll-like receptors) pathways and responsiveness to interferon [51], [52]. The overexpression of miR-150 promoted the proliferation and growth of gastric cancer cells by directly regulating the pro-apoptotic gene EGR2 (early growth response 2) and of lung cancer cells by targeting P53 [53], [54]. miR-204 could maintain epithelial barrier function and cell physiology by directly targeting TGF-β receptor 2 [55]. miR-132 and miR-212 belong to the same cluster and show considerable overlap in predicted target genes. Expression of the two miRNAs has a critical role in the proper development, maturation and function of neurons. And their abnormal expression is associated with neurological disorders, inflammation and immue regulation [56]. miR-218 was considered as one of tumor suppressive miRNAs, and involved in modulation of the nuclear factor-kappa B (NF-κB) signaling pathway by directly targeting the IKK-β (IκBs kinase) gene and in activation of the mTOR (mammalian target of rapamycin)-Akt signaling pathway targeting the mTOR component Rictor [57], [58].

The GO analysis of the differentially expressed miRNAs illustrated that a high enrichment of GOs was involved in membrane-bounded organelle, single-organism cellular process, ion-binding, organic substance metabolic process and primary metabolic process, etc. Interestingly, GOs of response to stress, immune response, and antigen processing and presentation were also significantly enriched. The KEGG pathway analysis described that 21 pathways, which included cytokine-cytokine receptor interaction, cell adhesion molecules (CAMs), chemokine signaling pathway, hematopoietic cell lineage, viral myocarditis, PPAR signaling pathway, antigen processing and presentation and T cell receptor signaling pathway, and so on, were over-represented. The results revealed that almost all enriched pathways were involved in metabolisms, immune functions and stress responses.

In summary, our results enriched the known miRNA species and predicted the high number of novel candidate miRNAs in porcine intestinal tissue. Our study showed a distinctive miRNA expression profile in the small intestine of weaning piglets and suckling piglets, and the number of differentially expressional miRNAs was affected by days after weaning. The GO terms and KEGG pathway annotations of the predicted miRNA target genes further described the potential functions of the differentially expressed miRNAs during weaning stress.

## Materials and Methods

### Animals

Animal studies were conducted in accordance with the principles and guidelines of the Zhejiang Farm Animal Welfare Council of China and approved by the ethics committee of Zhejiang Academy of Agricultural Sciences. In this experiment, four newborn litters of crossbred piglets (DYL, originating from mating Duroc boars with Yorkshire-Landrace sows) and their respective sows were housed separately in pens with farrowing crates. The DYL pig has been widely used in modern swine industry, but it is prone to suffering from the adverse effects of weaning stress. The piglets had *ad libitum* access to water and ground pre-starter feed. A total of 24 piglets (six per litter; body weight, 7.15±0.17 kg each) were removed from the four litters at 25 d of age. In this experiment, 12 piglets (3 per litter) were randomly selected and assigned to the weaning group at 25 d; they had *ad libitum* access to water and commercial pig diet. The remaining 12 piglets, which were assigned to the control group, were allowed to continue suckling with their sows.

### Sample Preparation

In this experiment, four piglets (one per litter) from the weaning group at 1, 4 and 7 d after weaning (libraries W1, W4, and W7, respectively) were sedated with xylazine and ketamine and euthanized with an overdose of pentobarbital administered via an ear vein. Similarly, four piglets from the control group at 26, 29 and 32 d of age (libraries S1, S4, and S7, respectively) were sedated and euthanized. The abdominal cavity of the piglets was opened, and intestinal samples were removed from the jejunum as described by Scholven et al. [59]. All intestinal samples were frozen in liquid nitrogen and stored at −80°C.

### Total RNA Isolation from Intestinal Samples

To obtain representative measurements from the 2-cm jejunal samples, three cross-sections of approximately 2 mm were pooled and lysed using PRO 200 Post-Mounted Laboratory Homogenizer (PRO Scientific, Oxford, USA). Total RNA was extracted using an E.Z.N.A. HP Total RNA kit (Omega Bio-Tek, Norcross, USA). Total RNA concentration was determined in a NanoDrop1000 spectrophotometer (Thermo Fisher Scientific, Wilmington, USA). RNA quality was assessed by electrophoresis on a formaldehyde-containing, ethidium bromide-stained 1% (w/v) agarose gel. The 28S/18rRNA ratio was quantified.

### Small RNA Library and Solexa High-throughput Sequencing

For small RNA library construction, 4 jejunal total RNA samples isolated from each treatment and control were separately pooled with equal contribution. The total RNA isolated from the weaning and control groups generated six small RNA libraries: W1, W4, W7, S1, S4 and S7. Solexa high-throughput sequencing was performed; 10 µg of total RNA was size-fractionated with Novex 15% TBE-Urea gel (Invitrogen, Karlsruhe, Germany), and RNA fragments (15–32 nt) were isolated. The purified RNA fragments were ligated with 5′ adapter (Illumina, CA, USA) and 3′ adapter (Illumina) using T4 RNA ligase (Ambion, Austin, TX, USA). Subsequently, the RNA fragments with the adapters at both ends were reverse-transcribed to single-stranded cDNA using Superscript II reverse transcriptase (Invitrogen, Carlsbad, CA, USA). The resulting cDNA was subjected to 15 PCR cycles using small RNA primer sets (Illumina). The amplification products were quantified in an Agilent Technologies 2100 Bioanalyzer (Agilent Technologies, Palo Alto, CA, USA) and sequenced in a HiSeq2000 sequencing system (Ilumina) at Genergy Biotech Co. Ltd. (Genergy, Shanghai, China).

### Processing of High-throughput Sequencing Data

Raw data were processed into clean data using Illumina’s Genome Analyzer Pipeline software, by removing all low-quality data, clipping 3′ adapters and 5′ adapters, discarding poly A/T containing sequences and fragments <15 nt and >32 nt. The remaining 15–32 nt clean data were used for the following analysis. All clean data were used to search the ncRNA to remove non-miRNAs (rRNA, snoRNA, snRNA,HACA-box, CD-box, Ribozyme, CRISRP, thermoregulator, IRES, scaRNA) with the Rfam10.1 and NCBI GenBank databases.

### Known and Novel miRNA Candidate Precursors

The clean data were mapped to the *Sus scrofa* draft genome assembly (SGSC Sscrofa 9.2, NCBI project 10718, GCA_000003025.2) with no mismatches using the Bowtie program. Unique sequences were aligned with swine miRNA sequences from miRBase 19.0 to identify known swine miRNAs [60]. Novel miRNA candidate precursors were identified using the miRDeep method [61]; the secondary structure stability of miRNAs was predicted using RNAfold algorithms [62]. Briefly, known and novel miRNA candidate precursors were determined by discarding reads that mapped to rRNAs, tRNAs, snoRNA and scRNA, using sequence reads to excise potential miRNA precursors from the genome, discarding unlikely miRNA precursors and using miRDeep core algorithms to calculate probabilistic scoring of structure and signature.

### Analysis of Differentially Expressed miRNAs

Expression levels of miRNAs were quantified by FPKM values using the Cufflink software [63]. Differences in miRNA expression levels between the weaning and control groups were assessed by the normalization method. Analyses were performed using two-tailed Student’s t-test; significant differences were set at q <0.05 (q-value is the adjusted p-value of the test statistic).

### RT-qPCR

RT-qPCR of separate RNA samples used for sequencing was performed. The stem-loop RT-qPCR method was developed by Chen et al. [64] and used by other researchers [65], [37]. ReverTra Ace reverse transcriptase (Toyobo Co., Osaka, Japan) and miRNA-specific stem-loop RT primers were used to synthesize cDNA. The primer sets of U6 SnRNA, ssc-miR-9-1 and URP have been described elsewhere [37]. Reaction mixtures were incubated at 65°C for 5 min, 37°C for 15 min and 98°C for 5 min to inactivate reverse transcriptase. RT-qPCR was performed using SYBR Green Real-time PCR Master Mix (Toyobo, QPK-201) and ABI StepOne Plus real-time PCR system (Applied Biosystems, Singapore). Swine U6 snRNA was used as an internal control; all reactions were run in triplicate. Relative quantification was calculated using the 2^−ΔΔCt^ formula [66].

RT-qPCR data were analyzed by Student’s t-test, using the SPSS version 17.0 statistical software. The results were expressed as mean ± SE. Statistical significance was set at p<0.05.

### Gene Target Prediction and Functional Annotation

In this experiment, the TargetScans software was used to scan targets for all differentially expressed miRNAs between the weaning and control groups; the method has been described in detail by Lewis et al [36]. The thresholds for candidate target sites were an exact match to positions 2–8 of the mature miRNA, or to positions 2–7 of the mature miRNA with a downstream ‘A’ across from position 1 of the miRNA or to positions 2–8 of the mature miRNA with a downstream ‘A’ across from position 1 of the miRNA. Gene 3′UTR sequences from swine genomic data were obtained from the Ensembl database. GO terms were enriched by the GOEAST software, and GO functional enrichment of differentially expressed miRNAs was categorized using level 2 biological processes (q-value is the adjusted p-value of the test statistic) [67]. KEGG pathway annotations were performed using the DAVID gene annotation tool, significance was set at q<0.05 (q-value is the adjusted p-value of the test statistic).

The raw data and processed files for the six libraries in this publication have been deposited in NCBI Gene Expression Omnibus database and are accessible through GEO Series accession number GSE 50500.

## Supporting Information

Figure S1 **The length distribution of small RNA in the libraries.**
(TIF)

Figure S2 **Pie chart for total reads matched to Rfam database.**
(TIF)

Figure S3 **Pie chart for unique reads matched to Rfam database.**
(TIF)

Table S1 **Reads obtained from Solexa high-throughput sequencing of small RNA fragments.**
(DOC)

Table S2 **Read counts mapped to** ***Sus scrofa*** **genome and located on chromosomes.**
(XLS)

Table S3 **Sequences matched to the** ***Sus scrofa*** **genome.**
(XLS)

Table S4 **Clean reads and clean unique reads matched to miRbase.**
(DOC)

Table S5 **MiRNA precursors identified in the libraries.**
(XLS)

Table S6 **Mature miRNAs identified in the libraries.**
(XLS)

Table S7 **Distribution of read counts and species of known miRNAs identified in each library.**
(XLS)

Table S8 **Mature miRNAs derived from the 5 p arm and 3 p arm of the same pre-miRNAs in the libraries.**
(XLS)

Table S9 **Read counts and species of novel miRNA candidate precursors identified in the libraries.**
(XLS)

Table S10 **Differentially expressed miRNAs in weaning and control groups.**
(XLS)

Table S11 **Primer sequences used in real-time quantitative polymerase chain reaction (RT-qPCR).**
(XLS)

Table S12 **Predicted target genes for upregulated miRNAs in W4.**
(XLS)

Table S13 **Predicted target genes for downregulated miRNAs in W4.**
(XLS)

Table S14 **Gene Ontology (GO) functional enrichment for potential miRNA targets.**
(XLS)

Table S15 **Kyoto Encyclopedia of Genes and Genomes (KEGG) pathway annotations for potential miRNA targets.**
(XLS)
