# Supplementary figures and images for: MicroRNA Transcriptome in Swine Small Intestine during Weaning Stress
Source: PLoS One. 2013 Nov 18;8(11):e79343. doi: 10.1371/journal.pone.0079343 (PMC3832476; doi:10.1371/journal.pone.0079343)

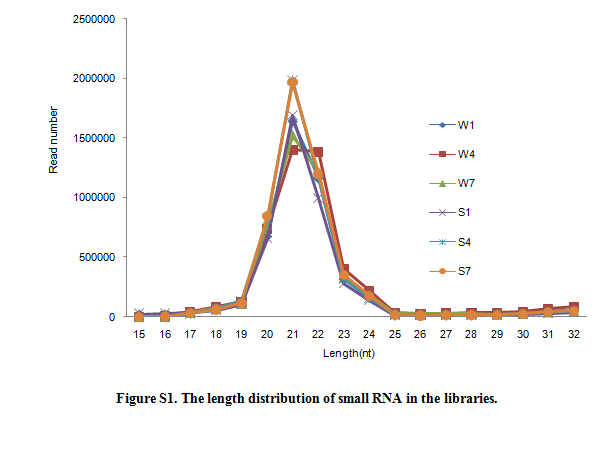

Supplement: Figure S1 — The length distribution of small RNA in the libraries. (TIF) [file pone.0079343.s001.tif]

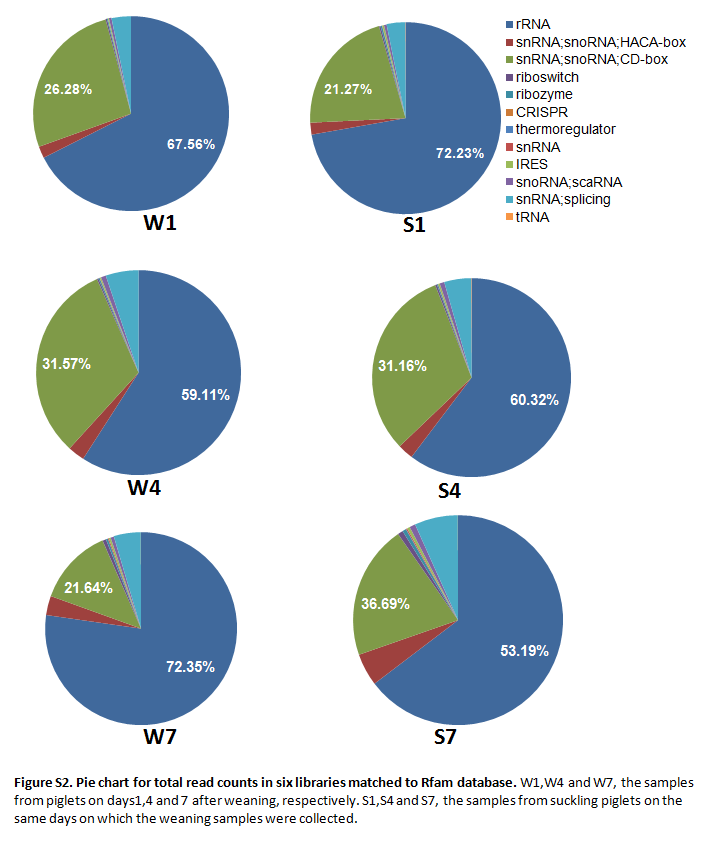

Supplement: Figure S2 — Pie chart for total reads matched to Rfam database. (TIF) [file pone.0079343.s002.tif]

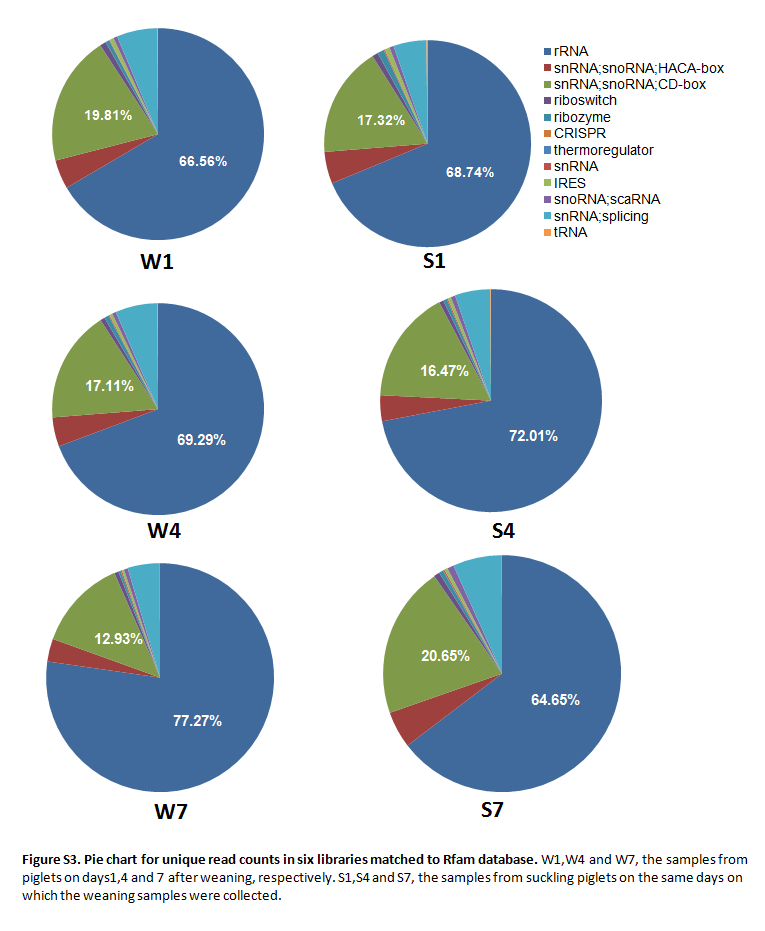

Supplement: Figure S3 — Pie chart for unique reads matched to Rfam database. (TIF) [file pone.0079343.s003.tif]
