# Supplementary material for: MicroRNA Transcriptome in Swine Small Intestine during Weaning Stress
Source: PLoS One. 2013 Nov 18;8(11):e79343. doi: 10.1371/journal.pone.0079343 (PMC3832476; doi:10.1371/journal.pone.0079343)
Supplement: Table S1 — Reads obtained from Solexa high-throughput sequencing of small RNA fragments. (DOC) [file pone.0079343.s004.doc]

**TableS1 The reads after solexa high-throughput sequencing of the small RNA fraction in six libraries.**

|  | Weaned treatment | | | | | |
| --- | --- | --- | --- | --- | --- | --- |
|  | W1 | S1 | W4 | S4 | W7 | S7 |
| Total reads | 5,273,249 | 5,021,654 | 6,409,389 | 5,916,236 | 5,531,307 | 6,031,126 |
| Reads trimmed 5’ adaptor | 198,569 | 152,459 | 449 | 266 | 316 | 193 |
| Reads trimmed 3’ adaptor | 3,858 | 3,552 | 3,370 | 4,393 | 2,611 | 3,076 |
| Reads trimmed low quality | 14,574 | 14,756 | 17,354 | 17,812 | 15,228 | 17,061 |
| Reads trimmed PolyA/T | 38 | 16 | 188 | 34 | 22 | 39 |
| Reads trimmed length  (<15nt or >32nt) | 609,698 | 650,256 | 1,623,028 | 997,606 | 1,017,461 | 1,069,240 |
| Clean reads | 4,446,512 | 4,200,615 | 4,765,000 | 4,896,125 | 4,495,669 | 4,941,517 |

W1,W4 and W7, the samples from piglets on days1,4 and 7 after weaning, respectively. S1,S4 and S7, the samples from suckling piglets on the same days on which the weaning samples were collected.
