# Supplementary material for: MicroRNA Transcriptome in Swine Small Intestine during Weaning Stress
Source: PLoS One. 2013 Nov 18;8(11):e79343. doi: 10.1371/journal.pone.0079343 (PMC3832476; doi:10.1371/journal.pone.0079343)
Supplement: Table S4 — Clean reads and clean unique reads matched to miRbase. (DOC) [file pone.0079343.s007.doc]

**Table S2 The clean reads and clean unique reads matched to miRbase in six libraries.**

|  | Treatment | | | | | |
| --- | --- | --- | --- | --- | --- | --- |
|  | W1 | S1 | W4 | S4 | W7 | S7 |
| Clean reads | 4,446,512 | 4,200,615 | 4,765,000 | 4,896,125 | 4,495,669 | 4,941,517 |
| Clean unique reads(hairpin) | 229,502 | 242,096 | 346,689 | 230,475 | 261,697 | 217,621 |
| Clean unique reads(mature) | 242,074 | 229,468 | 346,655 | 230,450 | 217,597 | 251,414 |
| Aligned reads(hairpin) | 3,409,847 | 3,180,950 | 3,529,620 | 3,816,407 | 3,834,504 | 3,481,565 |
| Aligned unique reads(hairpin) | 18,009 | 16,746 | 19,781 | 17,532 | 18,055 | 18,531 |
| Aligned reads(mature) | 3,206,994 | 3,005,674 | 3,323,403 | 3,607,372 | 3,284,848 | 319,285 |
| Aligned unique reads(mature) | 10,563 | 9,825 | 11,146 | 10,138 | 10,252 | 10,719 |

W1,W4 and W7, the samples from piglets on days1,4 and 7 after weaning, respectively. S1,S4 and S7, the samples from suckling piglets on the same days on which the weaning samples were collected.
